# A systematic review of context bias in invasion biology

**DOI:** 10.1371/journal.pone.0182502

**Published:** 2017-08-17

**Authors:** Robert J. Warren, Joshua R. King, Charlene Tarsa, Brian Haas, Jeremy Henderson

**Affiliations:** 1 Department of Biology, SUNY Buffalo State, Buffalo, New York, United States of America; 2 Biology Department, University of Central Florida, Orlando, Florida, United States of America; University of Hyogo, JAPAN

## Abstract

The language that scientists use to frame biological invasions may reveal inherent bias—including how data are interpreted. A frequent critique of invasion biology is the use of value-laden language that may indicate context bias. Here we use a systematic study of language and interpretation in papers drawn from invasion biology to evaluate whether there is a link between the framing of papers and the interpretation of results. We also examine any trends in context bias in biological invasion research. We examined 651 peer-reviewed invasive species competition studies and implemented a rigorous systematic review to examine bias in the presentation and interpretation of native and invasive competition in invasion biology. We predicted that bias in the presentation of invasive species is increasing, as suggested by several authors, and that bias against invasive species would result in misinterpreting their competitive dominance in correlational observational studies compared to causative experimental studies. We indeed found evidence of bias in the presentation and interpretation of invasive species research; authors often introduced research with invasive species in a negative context and study results were interpreted against invasive species more in correlational studies. However, we also found a distinct decrease in those biases since the mid-2000s. Given that there have been several waves of criticism from scientists both inside and outside invasion biology, our evidence suggests that the subdiscipline has somewhat self-corrected apparent biases.

## Introduction

How scientists conceptually frame biological invasions, and the language they use to do so, may suggest inherent bias in their approach [1]. Invasion science is a young field [2], initiated in the late 1950s [3] but really only flourishing as a distinct subdiscipline within ecology since the 1980s [4, 5]. Invasion biology publications hit the literature in force during the 1990s, and by the 2000s, controversy arose about bias in invasive species research [6–10]. Researchers suggested that the use of militaristic war language to vilify invasive species and apocalyptic descriptions of their potential impacts might, at best, exaggerate invasive species prowess and, at worst, suggest a lack of objectivity in invasive species research [8, 9]. The criticism of the field often came from disciplines outside invasion biology (e.g. historians of science, philosophers of science, [6, 11, 12]). These criticisms arose again, from within the field, in the 2010s as researchers suggested that invasive species research remains driven by a negative perception against invaders rather than empirical research [13, 14].

Experimenter predisposition can become an "unintended determinant of experimental results" even if the researcher is not cognizant of their own bias [15, 16]. For example, confirmation bias suggests that observers give greater weight to observations that confirm established beliefs or prejudice [16–18]. In medicine, context bias is a phenomenon whereby the prevalence and recency of observed disease influences subsequent diagnosis [19]. Finally, even supposedly empirical statistical results are subject to experimenter interpretation [20–22]. Many invasive species studies are correlative, comparing native and invasive species presence or abundance. Cause and effect in correlation go in either direction, or the observed effects may be caused by unmeasured, confounding variables (*Tertium quid* [21, 23]). Hence, a prejudice against invasive species, including the use of value-laden language in writing, could influence how research is designed and interpreted and produce results biased against them [1, 13, 24].

Given that some invasive species dominate ecosystems, presumably to the detriment of native species [25], many invasion biology hypotheses predict that invasive species outcompete native species because they are less constrained by enemies, bring superior "weapons" and rapidly evolve increased competitive abilities [26–29]. Whereas the assumption that invasive species are super-competitors that threaten the existence of native species is prevalent [13, 30], data supporting competition-based theories is mixed [31–34].

We examined 650 peer-reviewed invasive species competition studies to use the best-available evidence to investigate bias in the form of value-laden language in species invasion research and its temporal trends and correspondence with the interpretation of results. Our focus was a systematic review of *reported* competition in published research (we did not evaluate the study rigor or type of competition as our focus was on the research presentation and interpretation of results). Our objective was to investigate whether there exists a persistent bias demonstrated in invasive species research presentation and interpretation. Davis [1] and Thompson [13] noted that many scientific papers on invasive species start with 'boilerplate' text emphasizing generalized threats and impacts of invasive species rather than focusing on ecological theory, the study organism(s) or community at hand. Similarly, the Author Instructions from the peer-reviewed journal *Biological Invasions* implicitly address boilerplate introductions: “Please recall that *Biological Invasions* is read by specialists in invasion biology, so that introductory material pointing to the general importance of invasions is unnecessary and inappropriate.”

We examined the prevalence and trends in boilerplate introductions in invasive species literature and (1) we predicted that the phenomenon persists despite repeated criticisms of such language. Given that observational data are correlative and bidirectional, and experimental data are causative and unidirectional, we compared the reported results for competition from observational and experimental studies, and (2) we predicted that bias against invasive species would result in more frequently reporting their competitive advantage against native species in the observational studies.

## Materials and methods

### Literature search

**Fig 1:**
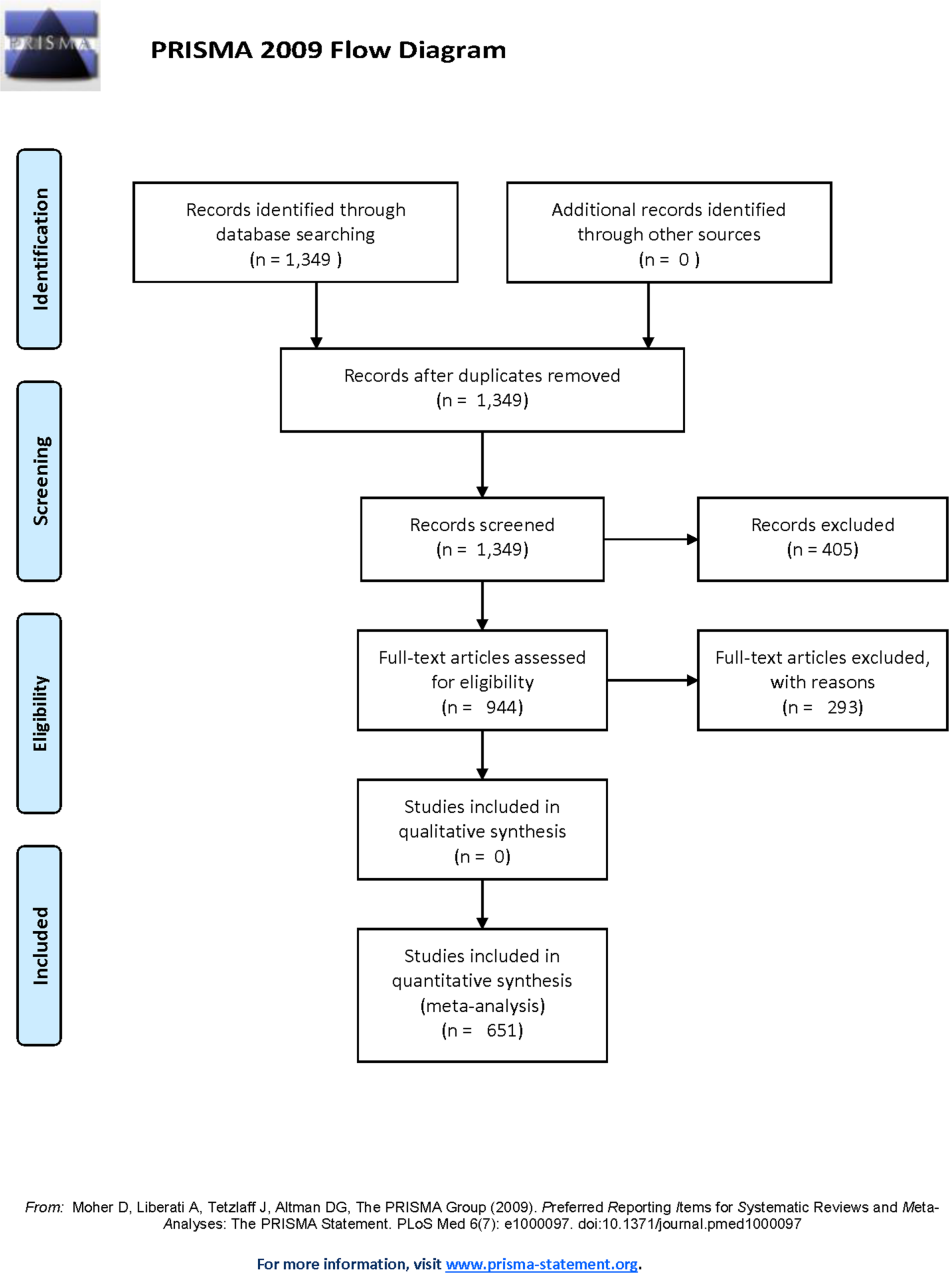
Flow diagram of the search and study selection.

We generated a list of peer-reviewed journal articles that assessed competition between invasive and native species using the search terms "invasive" * "native" * "competition" in the Web of Science database search engine September 2014. We did not place date or language restrictions on the search. We recognized that the terminology for exotic vs. non-native vs. invasive is unsettled (e.g., [13]), so we chose the common terms for their usage, both currently and historically. Whereas 'invasive' can connote a non-native species that is invasive, it also has become a general term for non-native species. Given that we used 'invasive' for our search term, we use the term throughout the paper. Indeed, the field itself is called "invasion biology." We used the default search engine settings for the Science Citation Index Expanded (1970-present) but excluded the Social Sciences and Arts & Humanities citation indices. We also refined the search by "article." The search produced a master list of 1,349 articles (Fig 1). We followed the Preferred Reporting Items for Systematic Reviews and Meta-Analyses (PRISMA) [S1 File].

A team of reviewers examined each journal article and screened out reviews, meta-analyses, simulations and non-English papers. We then did a more thorough reading of each paper and rejected those that did not test competition between invasive and native species or inferred or speculated about competition with no supporting evidence. We also conducted consistency tests whereby reviewers assessed the same set of articles and then met to compare results. We did not begin collecting quantitative review data until reviewer assessments were 90% in agreement. Our criteria narrowed the number of papers for quantitative analysis to 651. Reviewers also assigned each accepted paper a confidence weight (1–3) reflecting their confidence that it met the selection criteria: (1) Did not reject because the authors indicated competition, but not confident that the study appropriately tested or observed competition. (2) Intermediate confidence that the paper is a competition study. (3) Full confidence that the paper is appropriate for the quantitative review. We used the confidence ratings to weight the statistical analyses.

Reviewers coded the following data from each article: (1) boilerplate vs. theory/ecology–did the paper begin with a boilerplate overview of the negative impact of invasive species or introduce the biological/ecological background of the study? For example, Thompson [13] gave an example of boilerplate language in invasive species literature, ‴Many ecosystems worldwide are dominated by introduced plant species, leading to loss of biodiversity and ecosystem function'." (2) What type of organisms were studied (arthropod, avian, fish, mammal, plant, other)? (3) Was the system aquatic, riparian or terrestrial? (4) Was the study observational or experimental (or both)? (5) Was the study conducted in field or laboratory (or both) conditions? (6) If a field study, was the habitat natural (relatively intact) or anthropogenic (urban or human-altered environment)? (7) Was the reported better competitor the invasive or native species (or mixed, or no impact either way)? Studies with ambiguous or multiple impacts were excluded.

### Data analysis

The coded data were analyzed independent of the reviewer team so that the judgements used in analyzing the research papers were independent of statistical analysis. We analyzed the reported better competitor between invasive and native species using generalized linear models (GLMs) assuming a binomial error distribution using the R statistical package [35]. Studies that reported a positive outcome for the invader were considered 'successes' and those that reported a positive outcome for the native were considered 'failures'. We used the "car" package [36] in R to test for collinearity (variance inflation) in the models. We generated analysis of deviance (ANODEV) models for the fitted GLM models using Chi-square tests. The 'bias' ANODEV model included introductory emphasis (boilerplate, theory) and study type (experimental, observational) as factors. Variance inflation was < 1.2 in all models, and none were overdispersed (ϕ < 1.4).

Temporal trends in the use of boilerplate language introductions were analyzed using generalized least square (GLS) regressions with maximum likelihood. The GLS model assumes that errors are correlated and may have unequal variances without assuming linearity in the data. The model order (degree of autocorrelation) was selected using the Durbin Watson lag test [37]. We started with 1999 as it was the first year with *n* ≥ 10 studies. Trends in the reporting of invasive species as better competitors in papers that began with boilerplate and theoretical introductions, as well as experimental and observational studies, also were analyzed using GLS regressions. There were too few data points to use 1999–2000 for the observational data. For all models, we explored second order terms to test for non-linear trends.

## Results

We excluded 405 papers during the initial screening (reviews, meta-analyses, simulations and non-English papers) and then removed an addition 293 papers that did not assess competition between invasive and native species or provided no supporting evidence. Of the remaining 651 papers, we found that most studies reported invasive species as a superior competitor to native species (53.8%), whereas few reported native species as better competitors (7.5%). In many studies, however, the results were mixed (28.9%), and some studies did not find an appreciable effect either way (9.8%). More than half the study organisms were plants (57.9%), followed by arthropods (16.0%). Birds, fish and mammals together only made up 11.9% of the study organisms. 'Other' organisms comprised 14.2%. Terrestrial ecosystems were the most common study systems (64.9%), followed by aquatic ecosystems (26.5%), including riparian areas (8.3%). Most studies were conducted in the field (55.5%), 33.4% in a lab/greenhouse setting, and 11.1% using a combination of both. Most of the studies were experimental (73.8%), based on some level of variable manipulation, and 26.2% observational. Reviewer confidence was high that most (62.6%) of the papers met the criterion for the quantitative review, 28.6% were of intermediate confidence and 8.8% were of low confidence.

**Fig 2:**
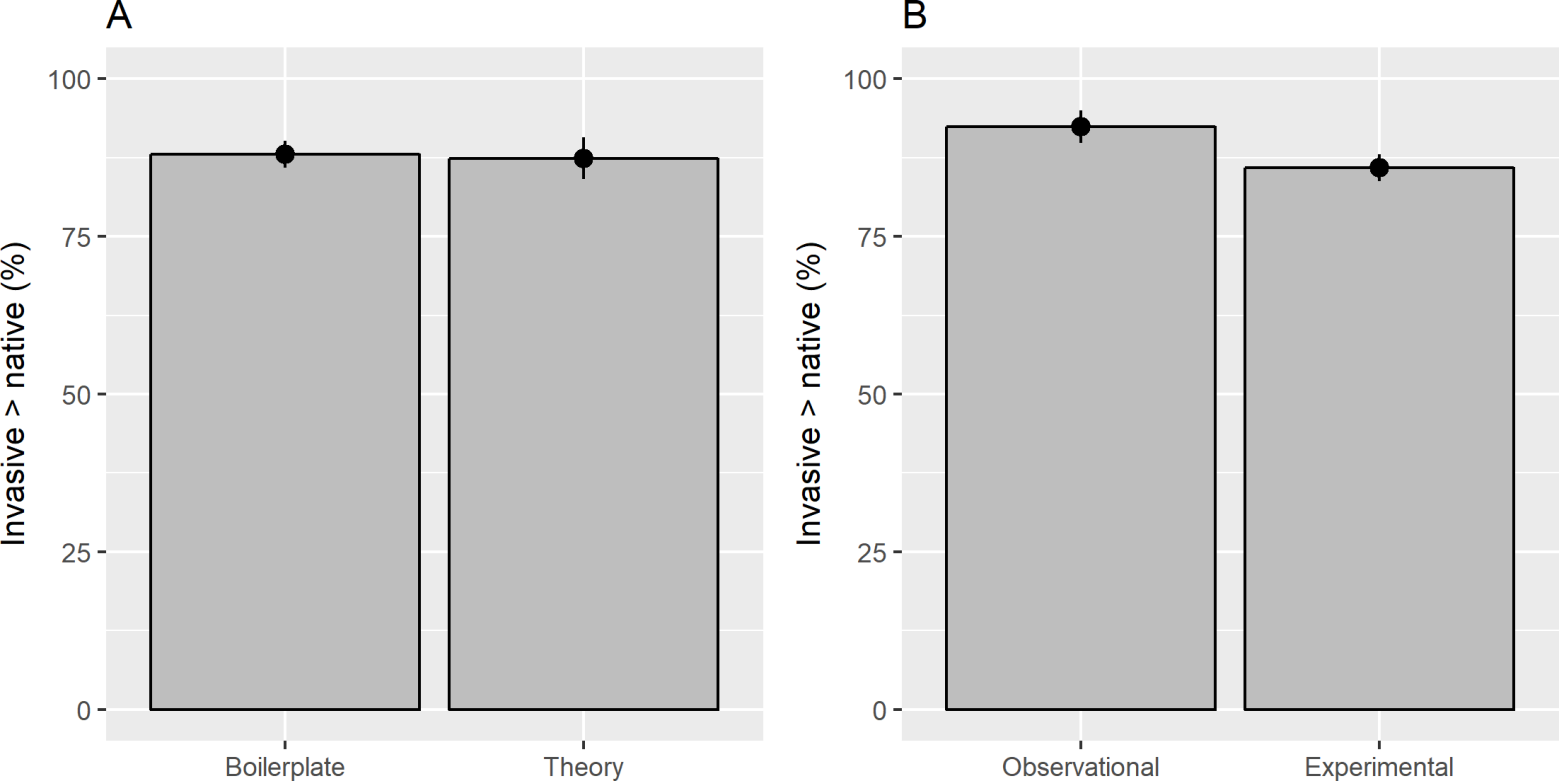
Published studies reported invasive species as better competitors than natives equally between those that began with a 'boilerplate' introduction placing invasive species in a negative light (a). Conversely, observational studies more often reported invasive species competitively superior than did experimental studies (b). Given that observational data are correlational and more subject to interpretation than experimental data, the discrepancy seemed to indicate interpretation bias against invasive species.

There was no significant difference (*df* = 1, *deviance* = 0.592, *residual df* = 334, *residual deviance* = 642.380, *p-value* = 0.441) in reporting invasive species as better competitors in papers that began with boilerplate (87.4%) or theoretical/ecological (88.0%) introductions (Fig 2A). However, invasive species were reported as superior competitors significantly more (*df* = 1, *deviance* = 6.896, *residual df* = 333, *residual deviance* = 635.480, *p-value* = 0.009) in observational (92.4%) than experimental (85.9%) studies (Fig 2B). Observational studies more often employed theoretical (97%) than boilerplate (88%) introductions.

**Fig 3:**
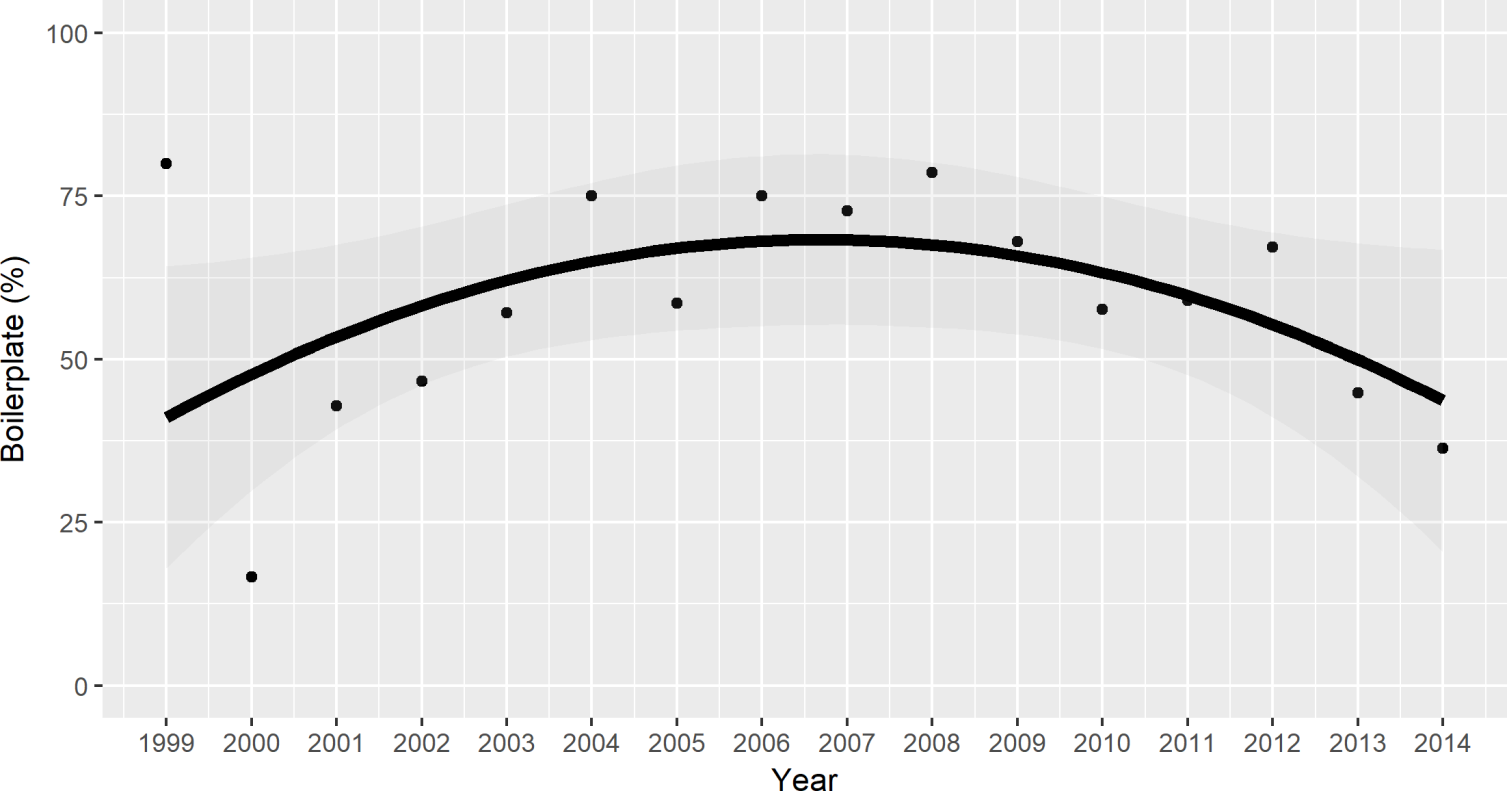
The percentage of invasive species papers that used boilerplate introductions peaked in the mid-to-late 2000s. This patterning may reflect the influence of several papers published 2003–2005 [6, 8–10] that criticized the use of negative and militaristic language in invasive species papers.

**Fig 4:**
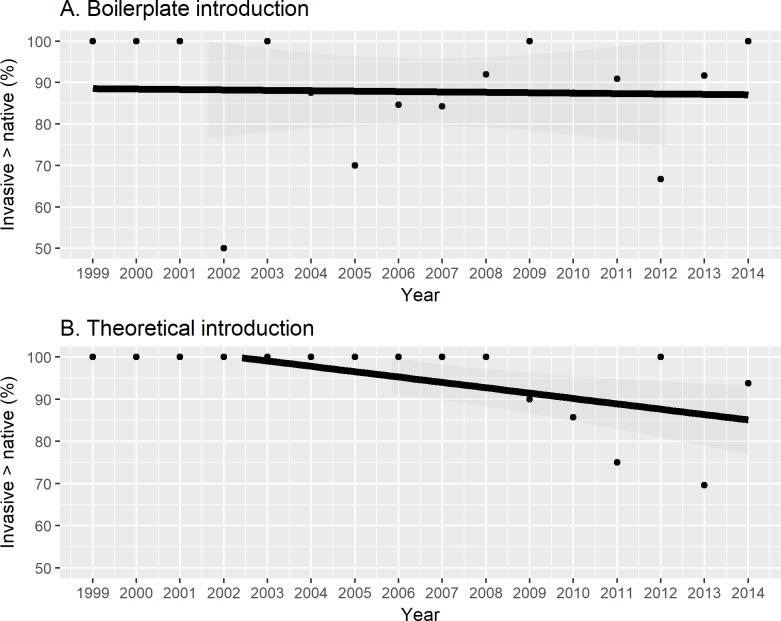
Papers that used a boilerplate introduction remained constant in reporting invasive species as better competitors than natives whereas those that using a theoretical introduction declined significantly in reporting invasive species as better competitors.

**Fig 5:**
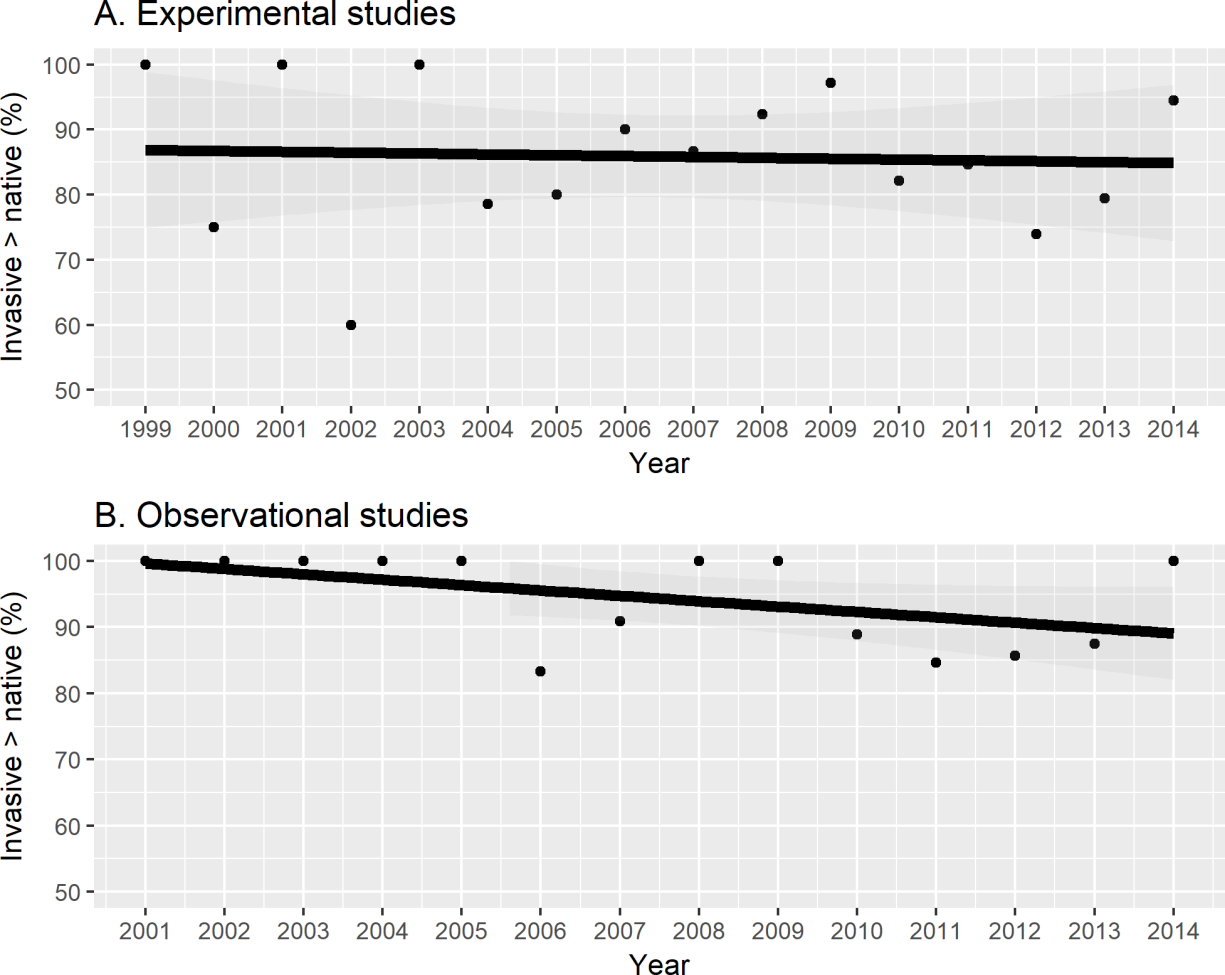
Experimental studies remained constant in reporting invasive species as better competitors than natives whereas observational studies declined significantly in reporting invasive species as better competitors. Observational studies generally are correlational, but experimental studies manipulate independent variables so that the results are less subject to experimenter bias in interpretation. As a result, the differences between observational and experimental studies on the same study subject may indicate research bias.

Time-series analysis indicated a significant long-term trend in using boilerplate introductions that peaked around 70% in the intermediate years between 1999–2014 (first-order, *coef*. = 2435.146, *SE* = 486.469, *t-value* = 5.005, *p-value* < 0.001; second-order, *coef*. = -607, *SE* = 0.121, *t-value* = -5.005, *p-value* < 0.001) [Fig 3]. The percent of boilerplate-initiated studies reporting invasive species as better competitors dropped, but not significantly, between 1999–2014 (*coef*. = -0.001, *SE* = 0.006, *t-value* = -0.153, *p-value* = 0.880) [Fig 4A]. However, the percentage of theory-initiated studies reporting invasive species as better competitors decreased significantly between 1999–2014 (*coef*. = -0.014, *SE* = 0.004, *t-value* = -3.664, *p-value* = 0.003) [Fig 4B]. The percentage of experimental studies reporting invasive species as better competitors did not change significantly between 1999–2014 (*coef*. = -0.001, *SE* = 0.003, *t-value* = -0.275, *p-value* = 0.787) [Fig 5A]. The percentage of observational studies reporting as better competitors decreased significantly between 2001–2014 (*coef*. = -0.009, *SE* = 0.002, *t-value* = -5.293, *p-value* < 0.001) [Fig 5B].

## Discussion

A frequent critique of invasion biology has been the use of value-laden language broadly framing invasive species as ‘harmful’ or ‘bad’ [1, 10, 12, 38, 39]. We hypothesized that such language is increasing in the introductory text published invasion biology research, but instead found a negative trend in 'boilerplate' language suggestive of bias in interpreting invasive species impacts. The data did support our second hypothesis, however, that context bias would emerge in observational studies with invasive species interpreted as better competitors significantly more than in experimental studies. Overall, our results suggested that context bias existed in the invasion biology papers we analyzed, but the bias decreased in recent years, suggesting some self-correction.

Context/experimenter bias may create an expectation of negative impact so that researchers and readers expect the worst of invasive species [9, 14]. We hypothesized that such bias might play out in studies that begin with a boilerplate introduction that focuses on the negative impacts of invasive species rather than focusing on their biological/ecological background. If this boilerplate approach indeed biased the researchers, then we expected they would report invasive species as better competitors than natives more often than those papers without a boilerplate beginning. Most (~68%) of the invasive species papers initiated with a boilerplate beginning, but they did not appear biased overall when compared to papers that initiated with an organismal or theoretical beginning. However, when analyzed across time, we found a trend in using boilerplate introductions that peaked in 2006–2007 (~75% of papers) and then declined until 2014 (~45% of papers). Moreover, reporting invasive species as better competitors remain constant in boilerplate-initiated papers whereas the same trend decreased significantly in theory-initiated papers. These results suggest that reduced use of biased language may coincide with increased data-driven, pluralistic views of invasive species interactions with natives. Nevertheless, the use of boilerplate language suggests that pre-conceived, assumptive views may persist, and there remains room for improvement

Given that observational studies are highly susceptible to biases in interpretation [40], we predicted that a second indicator of bias would play out in a difference between observational and experimental studies in whether they reported invasive species or natives as better competitors. And indeed, observational studies reported invasive species ~7% as better competitors more often than experimental studies. Moreover, in experimental studies, which are less interpretable and hence we would expect less room for self-correction, reports of invasive species as better competitors remained constant 1999–2014. However, in observational studies, which are much more subject to interpretation, reports of invasive species as better competitors dropped significantly 1999–2014. These results suggest some degree of self-correction. Where we observed shifts in invasive species reporting, these shifts appeared to begin in the late 2000s. A potential explanation for the shifts in invasive species sentiment was a cluster of publications questioning anti-invasive species language in 2004–2005 [8–10, 41]. In these papers, researchers argued for less outright disdain and language vilifying invasive species. They suggested treating invasive species like ecological experiments rather than enemies needing defeat. A holistic book critiquing the field [1], and a second wave of criticism aimed at boilerplate introductions and anti- bias against invasive species emerged toward the end of this period [13, 14], but our data suggests that biased language usage already had decreased by that point.

This systematic review did not assess the quality or efficacy of invasive/native species competition studies but only assessed whether the authors reported results as competition. Several systematic reviews have included in-depth analyses of plant competition research and experiments [42–46], whereas our focus was a systematic review of *reported* competition in published research regardless of organism. We used the search term "invasive species" which, in itself, may bias the results toward more aggressive exotic/non-native species. However, we implemented several measures to ensure empiricism in the review, including rigorously standardizing the quantification of papers, weighing the quantification to reflect reviewer confidence and separating the quantification process (and personnel) from statistical analysis.

We did not account for file drawer bias (unpublished null or non-significant findings), which certainly would influence findings if there is a bias against invasive species (studies showing no invasive species impact may be less publishable or perceived to be less publishable). We note, however that we did find a high percentage of papers that reported either mixed or no results (38.7%), which suggests that ‘negative’ findings were published. Methodological bias may exist in the studies themselves as the selection of robust invasive species and passive natives species as study targets may exaggerate the former's competitive abilities [24, 45]; However, our systematic study was relative to the papers published as we sought a bias signal between reported results and study approach.

Calls for the end of invasion biology as a separate discipline from ecology [1, 47] have prompted rigorous defense of the field [2, 48]; a field that often focuses on the eradication of invasive species and a return to a natural, native baseline [49]. Indeed, questioning the scientific validity of this invasion boilerplate has become somewhat a 'third rail' [50] with questioners castigated as scientific deniers of consensus [51]. We set out to test the claims of the skeptics, mainly that invasion biology papers often employ biased language and may themselves be biased against invasive species. We found signals of bias in invasive species research that seems, for the most part, to be slowly self-correcting, although bias clearly persists. Certainly, the general solutions for scientific integrity such as replication of invasive species studies and rigorous peer review likely have corrected, and will continue to correct, biases in invasion biology. However, we emphasize that rigorous self-criticism, such as that provided by several authors in the invasion biology field [6–10] may have produced aneffect in correcting inherent biases as the assumption that science is passively self-correcting may be folly [52, 53]. Defensiveness in a specific discipline, or science in general, is unscientific as the "first principle is that you must not fool yourself–and you are the easiest person to fool" [54]. In the end, a larger implication of these findings is to question science advocacy itself [55]. Judging invasive species as a scourge that all scientists must unanimously agree to stomp into oblivion seems a march toward surrendering data-driven science to advocacy, allowing reason become "a slave of the passions" [56].

Our results suggest that invasive species are indeed better competitors than native species, in agreement with other reviews and meta-analyses [42–46]; however, our goal was not the assessment of invasive species but an assessment of potential bias and correction in the science of invasive species. We found that bias trends in invasion biology appear to be decreasing, but roughly half the invasion biology papers still begin with a boilerplate introduction, and authors still interpret invasive species with greater negativity in correlative studies. In the invasion biology tempest, these results are equivocal, a projective test for interpretive biases themselves. That is, if one perceives invasion biology as a consensus that must be defended, the mere suggestion of biases will seem an attack. On the other hand, for those that perceive invasion biology as a dysfunctional field that should be reconsidered as a stand-alone subdiscipline of ecology, the trends toward improvement may seem trite or insubstantial.

## Supporting information

S1 File Preferred reporting items for systematic reviews and meta-analyses 2009 checklist. (DOC)
